# The cardiovascular risk factors of the Roma (Gypsies) people in Central-Eastern Europe: a review of the published literature

**Published:** 2012-12-25

**Authors:** M Dobranici, A Buzea, R Popescu

**Affiliations:** * Cardiology Department, “Colentina” University Hospital, Bucharest; ** ”Carol Davila” University of Medicine and Pharmacy, Bucharest

**Keywords:** Roma people, gipsy, cardiovascular risk factors

## Abstract

**Background:** Estimated number of the Roma people in central-eastern Europe cannot be precisely appreciated, but official data suggest that in the 2004 they were approximately 4.2 million. At this time, there are few available data about the health status of the Roma people, mostly assessing genetic and infectious diseases, which reflect poverty, overcrowding, and lack of education. There is even less data regarding non–communicable and chronic diseases, especially cardiovascular diseases.

**Methods:** We searched the published literature on the cardiovascular risk factors in Roma people using PubMed from January 2000 to July 2011. The searching criteria were: (1) randomized, prospective observational, retrospective and meta-analysis; (2) adult patients with cardiac diseases or cardiovascular risk factors (3) data available for cardiovascular patients. Search terms included dyslipidemia, obesity, tobacco, hypertension, and diabetes mellitus.

** Results:** Twenty-five studies were identified. Approximately 75% of them were related to just four countries: Slovakia, Croatia, Czech Republic, and Serbia. This paper is a review based on existing literature concerning classical risk factors in Roma people with emphasis on their ethnical features. Despite limited data, the results showed that this ethnicity has the incriminated risk factors more frequently than the majority and consequently a higher cardiovascular morbidity rate.

**Conclusions:** Quantification of the cardiovascular risk factor and their implication in the shortening of life expectancy in Roma population was a provocation due to a paucity of reliable data. At this time, we should pay more attention on the Roma health issues and the cultural concerns that might affect them in the context of borderless Europe.

## Introduction

The ancient Romany originate from India, and around 1300 they entered South-eastern Europe [**1,2**], then in 1350- 1400 in the Central and Western Europe. They were called “gypsy” because initially some peoples considered that they migrated from Egypt (E-gyptians) [**2,3**]. They had a poor life style, social problems and they were nomadic, and for all this, the normal populations developed a hostile attitude [**4**]. Throughout the 17–18th century, punitive policies were widely adopted, such as restrictions upon trade and shelter, prohibition of traditional dress or the speaking of Romani, and restrictions on Roma gatherings. Penalties included death and corporal punishment [**5**]. In the 19th century there was an improvement in their treatment in most of Europe, with the abolition of slavery and granting of legal rights, however the early 20th century is characterized by the theory of racial purity that contributed to the extermination of half a million Roma in Nazi camps [**6**]. In the post-war period, the conditions of Roma population improved in most of Europe. Their situation worsened after 1989 because all the problems were heightened by unequal unemployment and frequent negative interactions with authorities.

 Roma population health is thought to be a great unknown. The paucity of literature limits the scope for international comparison, and the small number of active researchers means that, for many countries, no information is accessible [**4**].

## Methods

Publications were identified as a result of a search of PubMed database starting from January 2000 until July 2011, using the keyword “Roma”. A total of 195 references were found mainly unrelated to the Roma people, but to the capital city of Italy. Another keyword used for searching was “Romani” and, for this term, we have found numerous articles with reference to the majority population of Romania. Unfortunately, this is a very frequent, unpleasant and sometimes unintentional confusion. For this reason, we tried some other alternatives: gipsy, gypsies, traveller, and cardiovascular risk factors in Roma people. The abstracts were manually assessed for the relevance to the Roma people. We excluded individual case reports, news reports and articles tagged “comment”. Most of the information was obtained from Slovakia, Croatia, Czech Republic, and Serbia. A few references were gathered from Hungary, Macedonia, Slovenia, Romania, and Bulgaria. 

## Results

The first question is related to the number of the Roma population. There are no reliable data about the number of the gypsy in Europe because they migrate and often they do not recognize their ethnicity [**7**]. Beside this, in other countries they were unrecognized as a minority for the purposes of census and in some other parts for unknown reasons. It is well established that they are socio-economically deprived, lack education, present a high rate of unemployment and overcrowding. An estimation of the European Commission of the Roma people in Central-Eastern Europe for the year 2002 is represented in **Table 1** [**8**].

**Table 1:** European Commission 2002: Estimated number of Roma people [8]

Country	Estimated number of Roma people
Bulgaria	700 000 – 800 000
Czech Republic	250 000 – 300 000
Hungary	550 000 – 600 000
Romania	1800 000 – 2500 000
Romania	480 000 – 520 000
Slovenia	6500 – 10 000
Latvia	8200

Turning to literature on health among the Roma people, there is a considerable variation in the number of citations from each country, which bears little relation to population size. A summary of the published literature concerning the cardiovascular risk factors in Roma people is resumed in **Table 2**. 

**Table 2:** Number of publications identified according to the country

Country	Number of papers identified
Slovakia	6
Croatia	4
Serbia	2
Slovenia	1
Czech Republic	1
Romania	2
Bulgaria	2
Macedonia	1
Hungary	2

Approximately 75% of the identified papers were related just to four countries, in this order: Slovakia, Croatia, Czech Republic, and Serbia. The subject areas will be examined in detail below.

Smoking is an independent major risk factor for coronary heart disease (CHD), cerebrovascular disease, and total atherosclerotic cardiovascular disease. The relationship between smoking and coronary heart disease, the effects of smoking on the atherosclerosis process, and the beneficial effects of smoking cessation are well known. Unfortunately, many smokers do not believe that smoking is harmful for them.

 Family history is a significant independent risk factor for coronary heart disease, particularly among younger individuals with a family history of premature disease.

Hypertension is a well-established risk factor for adverse cardiovascular outcomes, including CHD mortality and stroke. Systolic blood pressure is at least as powerful coronary risk factor as the diastolic blood pressure, particularly in older patients, and isolated systolic hypertension is now established as a major hazard for coronary heart disease and stroke. There is also evidence that the pulse pressure, which is determined primarily by large artery stiffness, is a predictor of risk.

 Lipids and lipoproteins. Epidemiologic data have documented a continuous, graded relationship between the serum total plasma cholesterol concentration and coronary risk. The following lipid and lipoprotein abnormalities are associated with increased coronary risk: elevated total cholesterol and LDL-cholesterol, low HDL-cholesterol, hypertriglyceridemia and increased Lp(a).

 Diabetes mellitus — Insulin resistance, hyperinsulinemia, and elevated blood glucose are associated with atherosclerotic cardiovascular disease. The 2002 National Cholesterol Education Program report designated diabetes a CHD risk equivalent, thereby elevating it to the highest risk category. In addition to the importance of diabetes as a risk factor, diabetics have a greater burden of other atherogenic risk factors than in non-diabetics, including hypertension, obesity, increased total-to-HDL-cholesterol ratio, hypertriglyceridemia, and elevated plasma fibrinogen. The CHD risk in diabetics varies widely with the intensity of these risk factors.

 Obesity — Obesity is associated with a number of risk factors for atherosclerosis, cardiovascular disease, and cardiovascular mortality. These include hypertension, insulin resistance and glucose intolerance, hypertriglyceridemia, reduced HDL-cholesterol, and low levels of adiponectin.

 Metabolic syndrome — Patients with the constellation of abdominal obesity, hypertension, diabetes, and dyslipidemia are considered to have the metabolic syndrome (also called the insulin resistance syndrome or syndrome X). Individuals with the metabolic syndrome have a markedly increased risk of coronary artery disease.

 For a better quantification of the available data, we analyzed the published literature separately for each country.

 Slovakia is the leading country in assessing the cardiovascular risk factors in Roma people. At the Department of Biology, Faculty of Humanities and Natural Science, University of Presov, Dolinska and collab. [**9**] have performed a relatively large sociological and biochemical-epidemiological study of gypsy population living in western Slovakia. The objective of the study was to obtain the health status data, which comprised of illness prevalence, blood pressure, obesity prevalence, complex biochemical parameters, immunology, nutrition manners, life style and different behavioural parameters of 345 of major population aged 18-60 years and to compare them with 269 Romanies. The results were statistically significant (p<0,05). In all age groups, the body-mass index (BMI) values of Gypsy women were higher than in those of non-Gypsy women. The Gypsy population has higher occurrence of obesity, overweight and hypertension, non-related to the region of country. However, in western Slovakia, the Gypsy minority has a higher prevalence of metabolic syndrome. The conclusion was that the risk of atherogenesis in Gypsy minorities is considerably increased and this is caused by unfavourable factors such as an increase in the prevalence of obesity, hypertension, smoking, and the deficiency in protective substances leading to dyslipidemia, hyperinsulinemia, cardiovascular disease, metabolic syndrome, and diabetes.

 De Courten B and collab. [**10**] have assessed the prevalence of type 2 diabetes mellitus (DM), metabolic syndrome and cardiovascular diseases in gypsies than in non-gypsies. They examined 156 Gypsies and 501 non-Gypsies who participated in a population survey. Age- and sex-standardized prevalence rates were computed for each of the following: type 2 DM, obesity, hyperlypidaemia, hypertension, hyperinsulinemia, elevated albumin/creatinine ratio (ACR), metabolic syndrome, and cardiovascular disease. The results were the following: age-sex standardized prevalence of type 2 DM was of 30% (95% CI=22-39) in Gypsies and of 10% (8-13, P=0.0001 for comparison of ethnic groups) in non-Gypsies. The corresponding prevalence of the other variables is the following: 65% (56-74) and 30% (26-34, P=0.0001) for obesity, 69% (61-76) and 59% (54-63, P=0.04) for hypercholesterolemia, 66% (59-74) and 39% (35-43, P=0.009) for hypertriglyceridemia, 49% (42-56) and 43% (39-47, P=0.1) for hypertension, 33% (26-50) and 8% (2-14, P=0.002) for hyperinsulinemia, 16% (9-22) and 5% (3-7, P=0.0001) for elevated ACR, 20% (12-27) and 4% (3-6, P=0.0001) for metabolic syndrome and 35% (28-43) and 26% (22-29, P=0.004) for cardiovascular disease. In conclusion, compared with non-Gypsies, Gypsies had a much higher prevalence of type 2 DM, metabolic syndrome and cardiovascular disease, which may contribute to their higher mortality [**10**].

 At the Department of Biology, Faculty of Science, Matthias Belius University, Hujova Z and al. [**11**] have evaluated cardiovascular disease (CVD) risk factors in relation to cigarette smoking in 174 Roma children and adolescents (88 males and 86 females) and 131 non-Roma individuals. In this bi-ethnic study, 26.4% of the Roma children and adolescents (more than twice contrary to the control group) were smokers. The most frequent CVD risk predictors of smoking Roma probands was low serum levels HDL-C, apo A (the Fisher test confirmed a significant relationship between cigarette smoking and HDL-C, apo A; p < 0.01)¹.

 In 2004, Krajcovicova-Kudlackova and collab. [**12**] assessed the lipid and non-lipid cardiovascular risk parameters (cholesterol, HDL- and LDL-cholesterol, triglycerides, homocysteine, C-reactive protein, insulin resistance) and data about blood pressure, smoking and BMI in two ethnic groups aged 19-35 years - the Gypsy group (n=122) and the Slovak group (n=137) of two regions with a high density of Gypsy population. In the Gypsy group, the values of triglycerides, atherogenic index, insulin, insulin resistance were significantly increased and the level of HDL-cholesterol was significantly decreased. The risk value of atherogenic index was found in 27% of Gypsy vs. 13% of majority subjects, and 28% vs. 24% of the subjects had hypertriglyceridemia. Risk value of insulin resistance (HOMA) was presented in 11% of the Gypsy vs. 5% of the majority group. More obese subjects (20% vs. 8%), more smokers (55% vs. 25%), and more subjects with low education (85% vs. 27%) were recorded in the minority group.

 In October 2010, Skodova and al. [**13**] published an assessment of differences in psychosocial factors and health-related quality of life (HRQL) between Roma and non-Roma coronary patients. They included 138 patients out of 437 interviewed: 46 Roma, all with low SES (socio-economical status), 46 non-Roma with low SES, and 46 non-Roma with high SES. Groups were matched for age, gender, and education. The GHQ-28 was used for the measurement of psychological well-being, the Maastricht interview for vital exhaustion, the type D questionnaire, and the Cook-Medley scale for personality and the SF-36 for HRQL. The results were that Roma people scored poorly compared to non-Roma in psychological well-being, vital exhaustion, and HRQL (p ≤ 0.001); however, these differences could be to a substantial extent explained by SES.

 In 2009, Simko V and Ginter E published an article [**14**] in which they have tried to explain the connection between short life expectancy and metabolic syndrome in Romanies (gypsies). The epidemiological and metabolic studies revealed in Romanies a high prevalence of obesity associated with an increased cardiovascular risk. There is no explanation for this seemingly paradoxical phenomenon, in a population living in poor economic conditions. It is possible that in the course of the many generation-long migration from India to Europe, pregnant Romanies and their foetuses suffered excessive nutritional deficiency. This might have induced adaptive metabolic and genetic changes aimed at an optimum utilization of scarce food supply. There is a hypothetical possibility that a “thrifty gene” was formed in them. The arrival of Romanies to Europe resulted in a somewhat better nutrition, along with a sharply reduced physical expenditure. The consequence is a metabolic syndrome with type 2 diabetes and increased cardiovascular mortality.

In 2009, Kolarcik and collab. assessed the occurrence of health-endangering behaviours among Slovak Roma adolescents in comparison to non-Roma adolescents and the impact of parental education and social desirability on the differences found. Among girls, Roma adolescents had lower rates of smoking, drunkenness and drug use than non-Roma (ORs from 0.14 to 0.60 compared to non-Roma), but had higher rates of physical inactivity. Among boys, drug use was less frequent among Roma adolescents (OR 0.12, 95% CI 0.03 to 0.46); differences for the other health-endangering behaviours were small and statistically insignificant. The effects of parental education and social desirability were small [**15**].

 There are fewer Romanies in the Czech Republic than in Slovakia. Last census in the Czech Republic, in 2001, estimated the minorities to represent only 4%, of that, Romanies only 0.11% (less than the Poles, Germans, Russians, and even Vietnamese). Some negative trends are linked to the lifestyle and health-related behaviour of Roma individuals. This is the case of smoking. Survey data clearly showed that anti-smoking measures, awareness campaigns, and debate of recent years did not have much of an impact on the lifestyle of the Roma population. Smoking is very widespread among Roma people. According to comparable data for the Czech population as a whole, the proportion of full-time smokers among the Roma ethnic group is several times higher (60% of Roma people, aged 16 and over, smoke every day and 10% are occasional smokers). Moreover, Roma are also heavy smokers and start very young, frequently during childhood (30% of today’s full-time smokers started at the age of thirteen or younger) [**16**].

Croatia has a substantial Roma population, the exact size estimated to be over 30 000, about 1% of the total population of Croatia. The Bayash are one of many Romani branches who, between the 14th and 19th centuries lived in the historical Romanian states Wallachia and Moldavia, where they were kept as slaves. After 1856, when the slavery in Romania was finally abolished, larger Bayash groups immigrated to Croatia.

 Zeljko Hand and collab. [**17**] state that in order to assess the health status and health-related lifestyle attributes of the Roma minority population living in Croatia, a multidisciplinary anthropological and epidemiological community-based study was carried out in 2005-2006 in the Bayash settlements in Baranja and Medimurje. They concluded that the prevalence of hypertension in the Bayash Roma is almost half of the magnitude of what is usually reported for the general population of Croatia. It is also lower when compared with the other European populations and this finding is not due to comparatively younger average age of the Bayash sample. The significant association of hypertension with age and BMI was confirmed in this study and the importance of non-traditional SES-related CVD risk factors were highlighted. Smoking is a part of traditional Roma life-style and, with 70% of smokers, almost the entire population is equally exposed to this risk factor in their family environment. Since it is homogenously distributed, this risk factor did not show to be a significant predictor of hypertension. In spite of the low prevalence of hypertension, the presented results are showing that Bayash Roma is bearing a high CVD risk factors load [**17**].

 In 2006, Zajc and al. [**18**] conducted a study that examined anthropometrically assessed nutritional status of the Bayash, the Roma population from the eastern Croatian region of Baranya, and compared it to the non-Roma general population of eastern Croatia. In general, the Bayash show low values of both primary anthropometric dimensions. Bayash women fall below the Croatian 10th percentile and men track about the 10th percentile. Both sexes approximate the 25th percentile for body weight. Despite their diminutive size, the Bayash appear to have adequate nutritional status until the age of 35 years after which their average BMI exceeds the value of 25 kg/m2 and falls in the overweight category. Their BMI ranges between the Croatian 50th and 75th percentile. However, 8% of the Bayash are underweight (BMI<18.5) in contrast to 1% of the majority population in the region. Underweight rates are especially high in women (11%) compared to men (4%). The prevalence of overweight (BMI 25.0 to 29.9) of 30% is considerably lower than in the majority population (42%) while the prevalence of obesity (BMI>or=30.0) of 23% is approximately equal. Overall unsatisfactory nutritional status of the Bayash deserves attention. It appears to be the product of unhealthy dietary habits and their socio-economic deprivation that resulted from their poor education and extremely high rate of unemployment [**18**].

 In 2009, Škarić-Jurić and collab. published an article “Genetic Risk Factors for Cardiovascular Diseases in the Roma Minority Population of Croatia” in the European Journal of Human Genetics (Vol. 17, Suppl. 2), in which they assumed that Bayash Roma population living in Croatia could be exemplary for having population-specific allele frequencies, including those for candidate genes for cardiovascular disease. Population analysis showed that the Bayash Roma population living in Croatia has a typical European frequency of the I allele of the ACE I/D polymorphism (45%), but the frequency of the allele 4 of the eNOS VNTR polymorphism (12%) as well as of allele G of the LEP G-2548A (34%) are lower than those found in the surrounding European populations, placing Croatian Roma population further east, among Asian populations. Present analysis showed that the Bayash Roma of Croatia, in comparison with other European populations, do not carry an increased genetic risk for cardiovascular diseases related to the most common polymorphisms of the ACE, eNOS and LEP genes [**19**].

 In 2010, Zeljko and collab. conducted a study in which the purpose was to examine whether the unique Bayash Roma genetic background exposed some new relations amongst well-known genetic and non-genetic CVD risk factors. The association between classical CVD risk phenotypes (hypertension, obesity and lipid status) and the four widely investigated CVD candidate gene polymorphisms was tested: ACE I/D, APOE (ε2, ε3, ε4), eNOS-VNTR (4,5) and LEP G2548A[**4-7**]. The strongest associations were found for ε2 allele of the APOE as a predictor of waist circumference (OR3.301; 95%CI 1.254-8.688; p=0.016) as well as for BMI (OR 3.547; 95%CI 1.471-8.557; p=0.005). It is notable that ε3 allele of APOE gene turned out to be a protective genetic factor determining low lipid levels [**20**].

 According to the 2002 Census, 0.2% of the inhabitants in Slovenia are Roma. However, based on re¬ports of the social centres and schools, their real number could be up to four times higher than this. The percentage of smokers among the Roma in Slo¬venia is unknown [**21**]. In 2005, Petek and collab. conducted a study to understand the reasons for widespread smoking behaviour among Roma in Slovenia, with the purpose of developing successful smoking cessation interventions. The content analysis revealed that smoking was a strong part of the cultural, ethnic, and individual identity of the Roma. Traditional strategies for smoking cessation are largely ineffective among the Roma because of their different attitudes towards smoking. Therefore, innovative and culturally acceptable methods need to be developed [**21**].

 According to the most recent population census in the Serbia from 2002, 108.193 or 1.44% residents declared themselves as Romani. In 2010, Beljić Živković and collab. investigated the prevalence of diabetes in the Roma population in Serbia. They screened 11 urban and 8 rural Roma communities (1465 Roma peoples) from October 2006 to May 2008 for the presence of diabetes. Blood glucose values, name, age, sex, presence of diabetes, family history, and obesity were recorded. Abdominal obesity was present in 600 (41%) participants. Eighty-seven participants (5.9%) already had diabetes and there were 76 (5.2%) newly discovered cases of type 2 diabetes. Participants with diabetes were significantly older (F= 28.33; P < 0.01). Family history for diabetes was positive in a third of the participants. The risk for diabetes was 3.48 times higher in participants with a positive family history (OR=3.47; 95% CI= 2.37-5.1; P < 0.01). Abdominal obesity was less frequent in healthy participants than in participants with diabetes (X2 = 32.55; P < 0.01). The risk of diabetes in participants with abdominal obesity was 2 times higher than in the non-obese (OR, 2.11; 95% CI, 1.24-3.55; P < 0.01). Diabetes was significantly more present in urban communities (X2 = 25.20; P < 0.01). The risk of developing diabetes was 3.65 times higher in participants from urban settlements (OR, 3.64; 95% CI, 1.99- 6.66; P < 0.01) [22].

 Bogdanović and collab. analyzed the mortality and population changes in the Roma and non-Roma population in Serbia in 2002 and 2005. The data show that mortality rates in the Roma population are significantly higher than in the general population, and morbidity structure of the most common causes of death significantly different from that of general population. Morbidity structure indicated that the most common causes of death in the Roma population were cardiovascular diseases. In relation to the general population respiratory system diseases were denoted as main causes of deaths in a significantly higher percent (6% vs. 3% in 2002 and 7% vs. 4% in 2005; p<0.001) and cardiovascular diseases in a significantly lower percent (44%:55% in 2002 and 46%:57%; p<0.001)[23].

 According to the official statistics of the National Statistics Institute (NSI), in 2001, the total number of the population in Bulgaria was of 7.928.901, of which 370 908 are Roma. These are the biggest ethnic communities in the country. Pioneers in the researches on Roma people are non-governmental organizations with the help of funding from non-state donors, mostly international organizations. At present, the topic of Roma exists in different studies, but the small number of them is devoted entirely to the health care. Although there are some individual studies of the health status of this minority group, the country does not conduct a systematic collection of health statistics by ethnicity [**16**]. Sabeva A. made a State of the Art Report for Bulgaria [**24**]. Almost two-thirds of Roma are young people and children. According to NSI, on the population census in 2001, the proportion of the Roma community of about 44% are up to 19 years old, while Bulgarians are under 20%. The proportion of elderly people over 60 years old in the Roma ethnic group is 4 times less than that of the Bulgarians. At present, the health status of Roma is characterized by negative high mortality, high morbidity, and low length of life. Average life expectancy of Roma is 10 years lower than the average for the country. The peak of the mortality of this group is between the 40-49th year, the most common reason for this being the cardiovascular diseases. Only 3% of Roma people live up to 60 years old. The main causes of deterioration of health of Roma are poverty, low education, unsanitary living conditions, unhealthy lifestyles associated with incomplete nutrition, constant distress and combined with other risk factors such as smoking, alcohol abuse, use of drugs, low motor activity. Access to health care for Roma is restricted by several factors such as poverty, remoteness of medical practices, particularly when a visit to a specialist is required, irregular payment of health insurance. Between a half and two thirds of the Roma feel that they are a victim of discrimination because of their ethnic origin or because of their poverty. A significant proportion of Roma is convinced that physicians and nurses are prejudiced against them because of ethnic and social reasons [**24**]. 

 In 2009, Masseria and colab. wrote an article [**25**] about the socio-economic determinants of the health status of Roma in comparison with non-Roma in Bulgaria, Hungary and Romania. They used a survey on Roma and non-Roma communities, provided by the United Nations Development Programme that includes socio-economic variables such as education, household expenditure, and wealth, as well as several health-related variables. Sample sizes are 2536 for Bulgaria, 2640 for Hungary and 3292 for Romania. People living in close proximity to the Roma community represent the non-Roma communities. On average, Roma people are more likely to be of younger age than the national majority population living in their close proximity. After adjusting for age and gender, the OR results showed that although demographic factors have had a significant effect on self-reported health status, between 2004 and 2003, Roma are significantly more likely to report a worsening of the health status than the majority population in all three countries. However, in Bulgaria, inequalities in health appeared more pronounced for other ethnic minorities than for the Roma. Inequalities in chronic illness and the perceived threat of illness were found between the Roma and the majority populations living in close proximity to the Roma, but with large differences across countries and by indicators of health. In Romania, the Roma are significantly more likely to report at least one chronic condition and to feel threatened by illness than the national majority of population, after adjusting for socio-economic and demographic determinants [**25**].

 Approximately 600.000 gypsies live in Hungary [**25**]. Gerevich J and collab. assessed the substance use of Roma compared with non-Roma adolescents in Journal of Nervous & Mental Disease (June 2010). Prevalence of tobacco and illicit drug use, and alcohol intoxication were examined in 225 Roma and 182 non-Roma adolescents. Results indicated a significant association between Roma ethnicity and a higher lifetime prevalence of tobacco use, alcohol intoxication, and illicit drugs use. Roma girls compared with non-Roma girls evidenced a disproportionately higher prevalence of smoking compared with the difference between Roma and non-Roma boys. Chi-square analyses showed for both Roma parents a higher level of tolerant attitude to smoking. The inequalities of the health status in substance use behaviours of the Roma versus non-Roma population, expressed in a more pronounced way in the female Roma population, emerge at an early age, based on their data; they are already observable in the early adolescent and adolescent age groups [**26**].

 According to the official census of the Republic of Macedonia from the year 2002, a number of 53.839 persons have declared themselves as Roma, which is 2,6% of the total population of 2.022.547. National Roma Centrum made a research to determine the present condition and top-priority problems regarding the access of Roma women to healthcare. Activities involved filling out forms and the study was conducted in 4 cities in the Republic of Macedonia: Kumanovo, Kriva Palanka, Prilep and Bitola, all with the purpose of getting a clear picture of the level of inclusion of Roma women in healthcare and the risk factors they are exposed to. The interviews included only females older than 13 years, representing 23,02% of the Roma population that lives in these four cities in the Republic of Macedonia. Based on the analysis of the persons interviewed about the presence of illnesses, which can be partly interpreted as partial and partly as medically accurate presence of an illness, the greatest presence is that of cardiovascular diseases. 440 of the interviewed persons said that they had heart trouble and blood pressure problems. It is an interesting fact that the 83 women from Kriva Palanka who said that they have problems with blood pressure, regulate their low blood pressure by consuming more liquids and salt [**27**].

 The Roma people (known as well as rromi or tigani) represent one of the largest ethnic minorities in Romania. According to census in 2002, there were 535.140 Roma people, representing 2,5% of the total population, being the second ethnic minority population after the Hungarian one [**28,29**]. The report „Sănătate şi comunitatea romă. Analiza situaţiei în România” was based on the descriptive and explanatory research whose ambitious objective was to analyze the health status in the Roma community, in order to prevent the inequalities and to elaborate specific political plans and activities for this target population 30. This report was realized while being based on the project „Health And The Roma Community, Analysis Of The Situation In Europe”, which is simultaneously implemented in six countries by organizations whose objective is the status improvement of Roma communities: Health of Roma Foundation in Bulgaria, Office of the Council for Roma Community Affairs (RVZRK) in Czech Republic, EFXINI POLI in Greece, Rede Europeia Anti Pobreza (REAPN) in Portugal, Centrul Romilor pentru Intervenţie Socială şi Studii (Romani CRISS) in Romania, Partners for Democratic Change (PDCS) in Slovakia [**16-25**]. The report was based on a quantitative type research by means of interviews made at the respondents’ home. The population was made up of 2616 subjects, 48,8% (1275) men and 51,2% (1341) women. The incidence of auto-declared chronic diseases was under 10% of the total population. The most frequent reported diseases were cardiovascular (hypertension, undocumented heart disease). The condition with the highest difference between gender is hypertension, 14,2% females vs. 6,3% males.

 For the classification of Roma population based on weight body-mass index (calculated using formula BMI = weight (kg) / height2 (m)) was used. Based on this index, the subjects were classified in categories normal, overweight, or obesity. 50,5% of the adults were in the normal category, whereas the minor population (under 18 years) with normal weight, represented 66,2%. In the overweight category 32,4% of adults and 17,3% of minors were included. The criteria for obesity was met in 17,1% of adult population, a bit above compared to the minor population in which the percent was of 16,6%. The gender analysis showed that 59,4% females 53,8% males are in the normal weight category. 23,9% females and 29,2% males were included in the overweight category. The obesity was equally met in females and males (16,7% and respectively 17%). According to the age, obesity was diagnosed in 23,6% of the population between 30 and 44 years old and 23,2% in those over 45 years old. The overweight incidence was higher in the population over 45 years old (37,1%), followed by the 30-44 years old subjects (32,4%). This report does not assert a specific pathology for Roma subjects who suffer from cardiovascular and nutrition diseases in same manner as the rest of the population.

In 2011, Bartos and Badila [**31**] assessed the prevalence of hypertension and other main cardiovascular risk factors in a gipsy population from Romania. They included 511 gipsy subjects. Prevalence of hypertension was of 30,3%, newly diagnosed hypertension 10%, number of hypertensive treated was of 49%; visceral obesity was of 50,8%, obesity BMI 34,4%, diabetes mellitus 10,3%, smoking 25,8%.

## Discussion

This review is subject to the limitations facing any literature review: incomplete ascertainment of papers and publication bias. Although literature overall is fragmentary, the most striking finding is the almost complete absence of research on non-communicable diseases. Several possible explanations exist but each has different implications. Some may reflect the difficulties of undertaking research in marginalized populations with well-developed sets of health beliefs. Classic risk factor epidemiology is difficult among Roma populations who, as well as being difficult to access, may regard researchers with hostility: often appropriately in view of their experiences with the authorities. In some societies, especially where strongly nationalistic sentiments have reappeared, evidence of health inequalities may be interpreted very differently than it might be in societies that are more inclusive. It is in the context of this climate that one must take a view on the appropriateness of different forms of intervention. While limited evidence exists, indicates that the health needs of the Roma population are considerable. The health status of the Roma population presents a major challenge to public health professionals, especially in some countries where they are a significant minority and where there may be discrimination, social exclusion, and even overt racism, not only because of the absence of research, but also due to the absence of advocacy on their behalf.

**Disclosures**

All authors: None to declare
